# Molecular Epidemiology of Rift Valley Fever Virus

**DOI:** 10.3201/eid1712.111035

**Published:** 2011-12

**Authors:** Antoinette A. Grobbelaar, Jacqueline Weyer, Patricia A. Leman, Alan Kemp, Janusz T. Paweska, Robert Swanepoel

**Affiliations:** National Institute for Communicable Diseases of the National Health Service, Sandringham, South Africa (A.A. Grobbelaar, J. Weyer, P.A Leman, A. Kemp, J.T. Paweska, R. Swanepoel);; University of Pretoria, Pretoria, South Africa (R. Swanepoel)

**Keywords:** Rift Valley fever, Rift Valley fever virus, viruses, molecular epidemiology, phylogenetics, evolution, Africa, Saudi Arabia

## Abstract

Phylogenetic relationships were examined for 198 Rift Valley fever virus isolates and 5 derived strains obtained from various sources in Saudi Arabia and 16 countries in Africa during a 67-year period (1944–2010). A maximum-likelihood tree prepared with sequence data for a 490-nt section of the Gn glycoprotein gene showed that 95 unique sequences sorted into 15 lineages. A 2010 isolate from a patient in South Africa potentially exposed to co-infection with live animal vaccine and wild virus was a reassortant. The potential influence of large-scale use of live animal vaccine on evolution of Rift Valley fever virus is discussed.

## Abstract 2

Large-scale vaccination of animals might have influenced virus evolution.

Rift Valley fever (RVF) is an acute disease of domestic ruminants in Africa and the Arabian Peninsula. This disease is caused by a mosquito-borne virus of the family *Bunyaviridae* and genus *Phlebovirus*. Large outbreaks occur at irregular intervals when heavy rains favor breeding of mosquito vectors of the virus and are characterized by deaths of newborn animals and abortion in pregnant sheep, goats, and cattle. Humans become infected by contact with tissues of infected animals or from mosquito bites and usually show development of mildly to moderately severe febrile illness. However, severe complications, including ocular sequelae, encephalitis, and fatal hemorrhagic disease, occur in some patients (*1*).

Rift Valley fever virus (RVFV) has a negative-sense, single-stranded RNA genome comprising large (L), medium (M), and small (S) segments. The L segment encodes viral RNA polymerase. The M segment encodes envelope glycoproteins Gc and Gn, a nonstructural protein, and a 78-kDa fusion protein of nonstructural and Gn proteins. The S segment shows an ambisense strategy and encodes nucleocapsid protein N and a nonstructural protein (*2*).

Early genetic analysis involved nucleotide sequencing of M segment RNA fragments encoding glycoprotein that induced neutralizing antibody response in 22 isolates obtained over 34 years in 6 countries (*3*). This analysis showed remarkable stability of sections of the genome expected to be under greatest immune selection pressure. The diversity observed in isolates from Zimbabwe indicated that outbreaks do not invariably involve a single genotype of virus but can result from intensified transmission of multiple strains already circulating in RVF-endemic areas. Nevertheless, neutralization of isolates with monoclonal antibodies confirmed that a single vaccine should suffice to control the disease (*3*). Slightly greater variability was observed among isolates when nonstructural protein sequence data were analyzed (*4*). Partial sequences determined for all 3 RNA segments of the genome of 20 isolates sorted into 3 geographically linked lineages associated with western Africa, Egypt, and eastcentral Africa and showed evidence of reassortment of genome segments between some sub-Saharan isolates (*5*).

High-throughput technology facilitated whole-genome sequence analysis of 33 isolates and resulted in confirmation of low genetic diversity of the virus and separation of the isolates into 7 lineages. There was no mutually exclusive correlation between genotype and geographic origin; representatives of geographic areas tended to cluster, but isolates from distant locations occurred in each lineage, indicating continuous widespread dispersal of virus (*6*). The remarkable congruence of phylogenetic trees for the 3 genome segments suggested that reassortment was not common, but convergence of some lineages within genome segments implied that reassortment had played an evolutionary role in the history of RVFV. Bayesian analysis suggested that the time of divergence of RVFV isolates from a most recent common ancestor dated to 1880–1890, the colonial period when the introduction of large concentrations of susceptible sheep and cattle would have facilitated exploitation of a new niche by an unknown progenitor virus. The evolutionary rate of the virus was similar to that of other RNA viruses. Thus, low nucleotide diversity probably relates to recent derivation from a common ancestor rather than stability of the genome.

Whole-genome sequencing and Bayesian analysis of 31 isolates associated with the 2006–2007 outbreak of RVF in Kenya showed that 2 sublineages of virus had evolved separately before or during a large outbreak during 1997–1998, with continued expansion of 1 sublineage dating from 2–4 years before 2006, confirming that outbreaks in disease-endemic areas might be associated with multiple lineages of virus, and that virus activity and evolution can occur below the threshold of detection by public health or animal authorities during interepidemic periods (*7*). Other genetic studies have been more limited in scope and concerned with either locating and investigating mechanisms of pathogenicity or determining phylogenetic relationships of isolates involved in particular outbreaks (*8**–**14*).

Analysis of partial M segment sequence data for a large collection of isolates and derived strains obtained from various sources in Saudi Arabia and 16 countries in Africa during 1944–2010 showed phylogenetic relationships not apparent in studies involving a smaller range of isolates. A 2010 isolate from a patient in South Africa potentially exposed to co-infection with live animal vaccine and wild virus from a needle injury while vaccinating sheep plus selected other isolates were subjected to limited sequencing of all 3 segments of the genome to obtain evidence of reassortment. We present and discuss the epidemiologic implications of these findings.

## Materials and Methods

### Viruses

Sequence data for 33 viruses were obtained from GenBank. The remaining 170 viruses for which we determined partial nucleotide sequences were obtained from the various institutions (Table A1).

### RNA Extraction

Viral RNA was extracted directly from 140 μL of infected human or livestock serum, clarified 10% organ suspensions, reconstituted freeze-dried mouse brain suspensions, or Vero cell culture supernatant fluids. Extraction was performed by using a QIAamp Viral RNA Kit (QIAGEN, Valencia, CA, USA) according to the manufacturer’s instructions.

### Reverse Transcription PCR and Nucleotide Sequencing of PCR Products

A 10-μL RNA aliquot was analyzed by using reverse transcription PCR with the Titan One Tube RT-PCR Kit (Roche Diagnostics, Penzburg, Germany) in a final volume of 50 μL as described (*15*). The forward primer FD1 (771/5′-CCAAATGACTACCAGTCAGC-3′/790) (*3*) and the reverse primer RVF E (1342/5′-CCTGACCCATTAGCATG-3′/1326) were selected to amplify a portion of the Gn glycoprotein gene of the virus; the primer positions correspond to the viral complementary DNA sequence of the M segment of the ZH501 human RVF virus isolate (*16*).

Amplicons were purified by using the Wizard PCR Preps DNA Purification System (Promega, Madison, WI, USA) according to the manufacturer’s instructions. Nucleotide sequences of PCR products were determined by using BigDye version 3.1 Terminator Cycle Sequencing Ready Reaction Kits (Applied Biosystems, Warrington, UK) according to the manufacturer’s instructions. For confirmatory purposes, sequences were obtained for both strands of PCR products by using primers FD1 and RVF E. Products were purified by using CentriSep spin columns (Princeton Separations Inc., Adelphia, NJ, USA) and analyzed by using a 377 GenAmp Sequencer and a 3130xl Genetic Analyzer (Applied Biosystems, Foster City, CA, USA).

On the basis of findings for M segment data, partial sequences were determined for S and L RNA segments of isolate SA184/10 from a patient potentially co-infected with live animal vaccine and wild virus; isolate SA54/10 from another patient in the same area; the batch of vaccine used (Smithburn neurotropic strain [SNS] 105/2010); SNS vaccine master seed virus; 95EG vaccine; and historical isolates 95EG Cow-2509, H1739, and H1825 (Table A1) to test for reassortment by using specifically designed primers. Primers F1 (1/5′-ACACAAAGACCCCCTAGTGC-3′/20) and R4 (1690/5′-ACACAAAGCTCCCTAGAGATAC-3′/1669) were used to amplify the S segment and the N gene (735 nt). Primers RVFL10 (4237/5′-GGTGTTGTGTCATCATTG-3′/4254) and L2 (4730/5′-GTGTGAGCTAGAGTTGCTTC-3′/4711) were used to amplify a 494-nt region of the L segment.

### Sequence Analysis

Nucleotide and amino acid sequence alignments were generated by using ClustalW Multiple Alignment analysis software as implemented in BioEdit version 7.0.5.3 (*17*). Unique sequences generated in the study were submitted to GenBank and assigned accession numbers indicated in Table A1. Preliminary phylogenetic analysis was performed by using a neighbor-joining distance method in MEGA4 that applied a Jukes-Cantor model under 1,000 bootstrap iterations (*18*). Sequence divergence was determined by using MEGA4 to calculate mean pairwise distances within groups. A phylogenetic tree was constructed for 95 isolates exhibiting unique sequences and for 16 isolates that exhibited duplicate sequences but that were isolated in different years or countries, by using the maximum-likelihood method in PAUP* version 4.0b2 (Sinauer Associates, Inc., Sunderland, MA, USA).

Further analysis of partial M segment sequence data was performed by using the Bayesian software package, which included BEAST, BEAUTI, Tracer, TreeAnnotator, and FigTree (*19*), by using a Markov Chain Monte Carlo chain length of 3.0 × 10^7^, a 3.0 × 10^6^ burn in, a generalized time reversible plus gamma plus invariant nucleotide substitution model, a relaxed uncorrelated logarithmic normal molecular clock, and sampling every 1,000 states.

To check for recombination events within the RVFV alignment, the alignment was examined by using a set of 6 detection methods implemented in RDP3 (*20*). Partial nucleotide sequence data for S, M, and L RNA segments of 33 isolates for which whole-genome sequences have been reported (*6*) (all included in Table A1) were obtained from GenBank and used with sequences determined in the present study for 5 isolates plus the 3 animal vaccine viruses to perform analyses as described above to test whether genetic reassortment had occurred.

## Results

**Figure A1:**
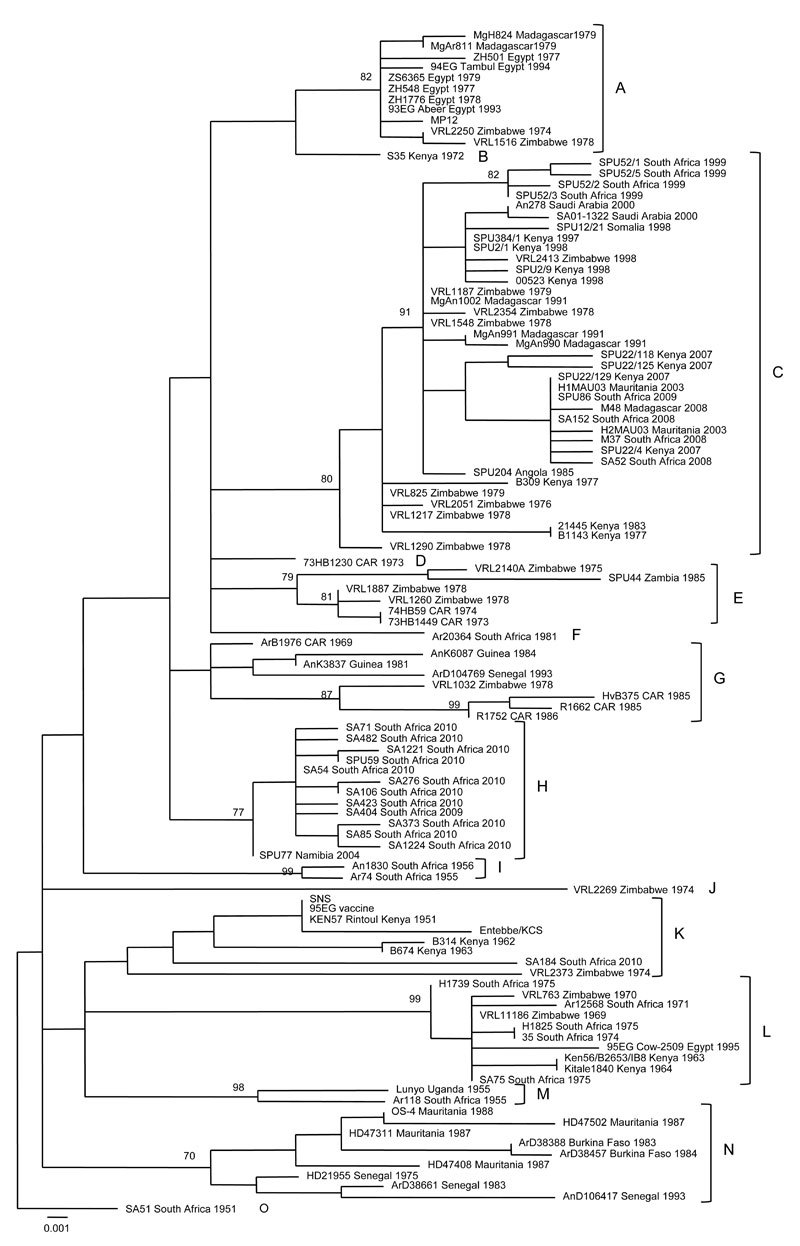
Maximum-likelihood tree for a 490-nt section of the Gn glycoprotein gene of 111 isolates and derived strains of Rift Valley fever virus from Africa and Saudi Arabia, 1944–2010. The 95 unique sequences sorted into 15 lineages (A–O). Mean pairwise distances (p-distances) were <0.017 within lineages, and bootstrap values were >70%. Scale bar indicates substitutions per site. CAR, Central African Republic; SNS, Smithburn neurotropic strain.

Diversity of partial M segment sequences was low; pairwise differences ranged from 0% to 5.4% for nucleotides and from 0% to 2.8% for deduced amino acids. These values are similar to those identified for 33 whole-genome sequences (*6*). Neighbor-joining, maximum-likelihood, and Bayesian phylogenies were similar. Bayesian analysis indicated that divergence of isolates included in the study from a most recent common ancestor dated to 1892, which is similar to the estimate of 1880–1890 deduced earlier from 33 whole-genome sequences. However, posterior support values for the tree nodes were weak because a small nucleotide segment was analyzed. Despite low genetic diversity, the 5 derived strains and 198 isolates of RVFV circulating over the past 67 years produced 95 unique sequences that resolved into 15 lineages (A–O) (Figure A1; Table A1) with mean pairwise distances <0.017 within lineages and bootstrap values >70% (*21*).

Five lineages (B, D, F, J, and O) contained single isolates, and lineage I contained 2 isolates from South Africa. Six other lineages (A, C, E, G, H and N) contained clusters of isolates associated with outbreaks in individual countries or regions, but each also included isolates from distant locations or separate outbreaks. Thus, lineage A comprised isolates from outbreaks in Zimbabwe in 1978, Madagascar in 1979, and Egypt in 1977–1978 and 1993 and a 1974 isolate from an interepidemic period in Zimbabwe (Figure A1; Table A1).

Lineage C was most widely distributed and contained isolates from major outbreaks in Zimbabwe in 1978, Madagascar in 1991, eastern Africa (Kenya, Tanzania, and Somalia) during 1997–1998, Saudi Arabia during 2000–2001, Kenya in 2007, and South Africa during 2008–2009 and lesser outbreaks and interepidemic periods in the same countries and Mauritania during 1976–2009. It was also associated with an isolated fatal infection in Angola in 1985 in a visitor from South Africa ostensibly exposed to mosquito bites (*22*). Lineage E, which was found in the Central African Republic and Zimbabwe during 1973–1978, was isolated from 2 patients referred to South Africa for treatment in 1985 after a relative had died of a similar disease in Zambia during an outbreak of RVF in livestock confirmed serologically (*22**,**23*). Lineage G contained isolates from the Central African Republic, Zimbabwe, Guinea, and Senegal during 1969–1986, and lineage H contained isolates from the 2009–2010 outbreak in South Africa and an apparent antecedent from Namibia in 2004. Lineage N, originally designated West African (*5*), contained isolates from Senegal, Burkina Faso, and Mauritania during 1975–1993.

Lineages K, L, and M with a common root node contained the SNS neurotropic and the KCS hepatotropic strains derived from the same mosquito isolate (*24*) and isolates from Kenya, Zimbabwe, South Africa, and Egypt during 1951–2010, countries that used the SNS animal vaccine on a large scale during major outbreaks of RVF. Isolate KEN57 Rintoul, obtained from a cow in Kenya in 1951, when the first batch of SNS vaccine was sent from South Africa to Kenya, had a partial M segment sequence identical to that of the vaccine virus. The SNS 105/2010 batch of vaccine and Egyptian 95EG vaccine also had the same sequence as the SNS vaccine master seed stock produced in 1987 (Table A1).

**Figure 1:**
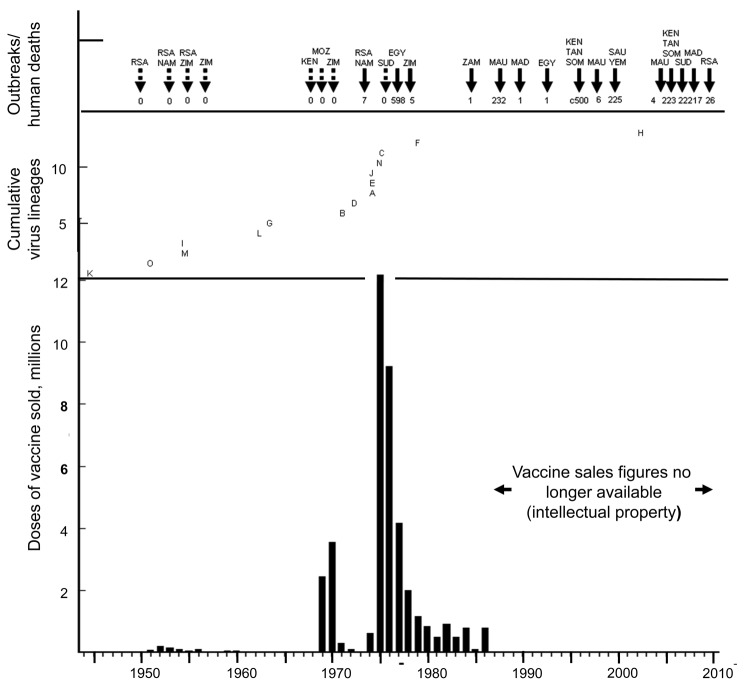
Annual sales of Smithburn neurotropic strain animal vaccine produced in South Africa in relation to cumulative viral lineages isolated and human deaths in major outbreaks of Rift Valley fever (RVF) in Africa and Saudi Arabia, 1944–2010. Broken arrows indicate RVF outbreaks without human deaths recorded, and solid arrows indicate RVF outbreaks with human deaths. RSA, Republic of South Africa; NAM, Namibia; ZIM, Zimbabwe; MOZ, Mozambique; KEN, Kenya; EGY, Egypt; SUD, Sudan; ZAM, Zambia; MAU, Mauritania; MAD, Madagascar; TAN, Tanzania; SOM, Somalia; SAU, Saudi Arabia; YEM, Yemen.

Isolate SA184/10, from a patient potentially exposed to wild virus and SNS 105/2010 animal vaccine, grouped with the parent vaccine strain in lineage K, albeit with a divergence of 11 (2.2%) of 490 nt (2 aa), and distantly from 46 other human isolates from the 2008–2010 outbreaks in South Africa. Moreover, isolate 95EG Cow-2509 from the fetus of a cow that aborted after the administration of 95EG vaccine in Egypt grouped in lineage L distantly from all other isolates from Egypt in lineage A (*12*) (Figure A1; Table A1). Isolates H1739 and H1825, which also grouped in lineage L, came from the first human deaths caused by RVF during a major outbreak in South Africa during 1974–1976 during which animal vaccine was used on an unprecedented scale (*25*; National Institute for Communicable Diseases [NICD], unpub data) (Figure 1). These 5 isolates were investigated for genetic reassortment.

Partial nucleotide sequence data for S, M, and L RNA segments derived from whole-genome sequences of 33 isolates produced phylogenetic trees with the same topologies as those published for the complete sequences (*6*). Isolate SA184/10 from the patient who sustained a needle injury while vaccinating sheep sorted with SNS vaccine virus in the M segment tree but with isolate SA54/10 in the other segments, and was clearly a reassortant. SNS 105/2010 and 95EG batches of vaccine corresponded with the SNS master seed stock for all 3 segments of the genome. Analysis of isolates H1739 and H1825 obtained from the first recorded human deaths caused by RVF in 1975 and isolate 95EG Cow-2509 from Egypt did not show clear-cut evidence of reassortment but fell into a group that manifested convergence of lineages D and E in the M RNA segment tree in the 33 whole-genome study, corresponding to lineages L plus K, the vaccine lineage, in the present study. This phenomenon was interpreted as evidence of historical reassortment (*6*). Isolate SA75 also ostensibly came from a human infected in South Africa in 1975 (*6*), but we could not relate the designation to records at NICD. Analysis of partial M segment sequence data did not show evidence of recombination.

## Discussion

Historical developments in RVF include spread beyond sub-Saharan Africa during 1977–2007 and fatal human infections during a large outbreak in South Africa during 1974–1976. Since that time, large outbreaks of the disease in livestock have invariably been associated with human deaths (*1*). Mechanisms for dispersal of RVFV fall beyond the scope of this report. However, refinements to phylogenetics, such as Bayesian-based and population-based genetic analysis, have shown that translocated virus does not necessarily arrive in receptive circumstances to trigger epidemics, but can initiate smoldering infection or seeding of the ground, which remains undetected until suitable climatic conditions precipitate outbreaks, as occurred ahead of the 2000–2001 and 2006–2007 outbreaks in the Arabian Peninsula and Kenya (*6**,**7*).

**Figure 2:**
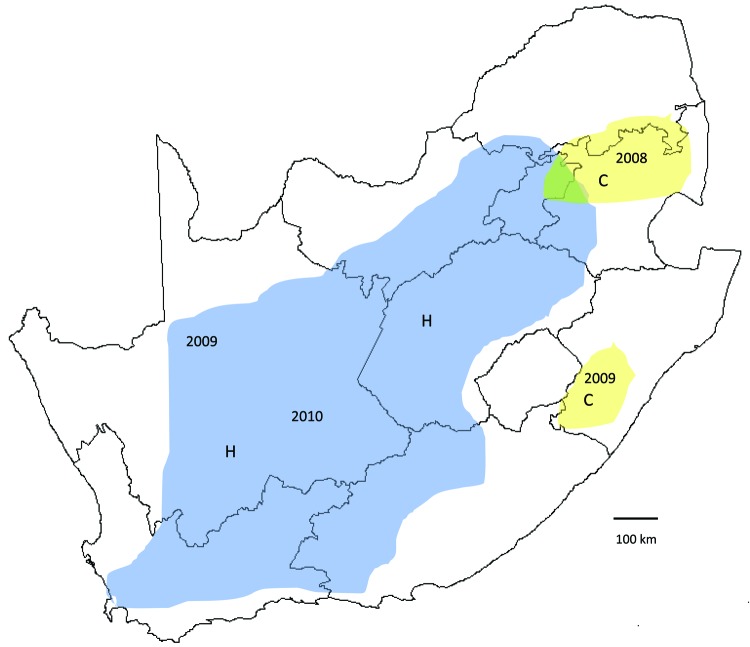
Recent outbreaks of Rift Valley fever in South Africa. Lineage C virus (yellow areas), which caused a small outbreak in Kruger National Park in 1999, was associated with scattered outbreaks of disease in adjacent parts of northeastern South Africa in 2008 and limited outbreaks to the south in KwaZulu-Natal Province early in 2009. Lineage H virus (blue area), which was first encountered in the Caprivi Strip of Namibia in 2004, caused focal outbreaks in the Northern Cape Province late in 2009, and was associated with coalescing outbreaks over much of interior South Africa in 2010. Lines indicate province boundaries.

Recent outbreaks in South Africa showed an analogous pattern. After heavy rains in 2008, lineage C virus, which had been isolated during a limited disease outbreak in Kruger National Park in 1999, was associated with scattered outbreaks of RVF in adjacent parts of northeastern South Africa. In the first half of 2009, the same lineage caused limited outbreaks to the south in KwaZulu-Natal Province. In the second half of 2009, lineage H virus, which had been encountered in the Caprivi Strip of Namibia in 2004, caused focal outbreaks in Northern Cape Province and was progressively identified in coalescing outbreaks over much of interior South Africa in 2010 (Figure 2). Diversity of genotypes observed among recent isolates of the 2 lineages (Figure A1), and results of our limited Bayesian analysis imply that RVFV had progressively reinfiltrated the interior plateau of South Africa during a period of increasing rainfall, 3 decades after the major outbreak of 1974–1976. There were 26 deaths among 244 persons infected with lineage H virus, and while no deaths were recorded in areas where lineage C virus was active, only 22 cases were diagnosed (NICD, unpub. data).

Reassortment between SNS vaccine virus and wild virus has implications for the safety of the vaccine and its possible role in evolution of RVFV. The SNS virus from Uganda (Table A1) was taken to mouse intracranial passage 102 and embryonated chicken egg passage 54 to produce avianized (decreased infectivity produced by repeated culture in chick embryo) animal vaccine in South Africa in 1951. After several adjustments, reversion was made in 1958 to SNS virus passed 103 times in mice only. Since 1971, the same virus has been propagated in BHK21 cells for preparation of freeze-dried vaccine (*24**,**26**–**29*). In Kenya, animal vaccine was initially produced from avianized virus from South Africa, but since 1960, SNS virus has been used at mouse passage 106 (*30**–**32*). SNS seed virus from South Africa was used to produce vaccine in Egypt in 1994, and South Africa and Egypt have used field isolates to produce inactivated animal vaccine (*12**,**27**,**29*). SNS vaccine is only partially attenuated, but accurate estimates for abortigenicity and teratogenicity were never determined because conflicting results were initially obtained in different sheep breeds and because pathogenicity varies with stage of pregnancy (*26**,**33*). However, there has been a tendency to regard risks associated with vaccine as acceptable in the face of an outbreak of RVF (*34*).

During 1951–1968, ≈1 million doses of SNS animal vaccine were sold in South Africa; sales were similar in Kenya (*28**,**32*). The vaccine was first used on a large scale in 1969–1970 when 6 million doses were issued, mainly to Zimbabwe, where a large outbreak occurred (*29*) (Figure 1). Sales decreased sharply by 1973 but increased to 22 million doses during the major outbreak in South Africa and Namibia during 1974–1976.

During the same period, 4.7 million doses of newly developed inactivated RVF vaccine and ≈14 million doses of Wesselsbron virus vaccine were sold (*27**,**28**,**35*). Wesselsbron is a mosquito-borne flavivirus initially thought to cause outbreaks analogous to RVF, and vaccine was produced by similar empirical attenuation of virus by intracranial passage in mice (*33**,**35*). Thus, >40 million doses of either vaccine were administered over 3 years in South Africa and Namibia, where the combined sheep, cattle, and goat population was <60 million. However, fewer than two thirds of the animals were in RVF-affected areas. Approximately 4.2 million doses of SNS vaccine were sold in 1977, much of it to South Africa, but also to Israel and Egypt. Approximately 3 million doses were sold in 1978–1979, mostly to Zimbabwe where an additional large outbreak of RVF had occurred. Subsequently sales remained <1 million doses per year until 1986, after which figures were no longer made public (*28*). However, substantial quantities of vaccine, mainly from South Africa, were used in eastern Africa and Saudi Arabia in recent years. In all locations except Israel and the Sinai Peninsula, where the threat of RVF was not proven, large numbers of animals were vaccinated only after onset of outbreaks (*28**,**29*).

Livestock vaccines are sold in multidose vials and commonly administered with automatic syringes and intermittent changes of needles. Vaccine is likely to be administered to some animals that are already infected. Thus, in view of intense viremia that occurs in RVF, serial transfer of wild virus by needle is a recognized hazard of vaccinating livestock during outbreaks (*29*). This vaccination likely resulted in co-infections with vaccine and wild virus, or even different wild genotypes, on multiple occasions over decades, particularly during 1969–1979, with an implied potential for the generation of recombinant and reassortant genotypes. Uptake and transmission of virus by vectors would have increased co-infections and potential for genetic interaction (*36*).

Although there is no corresponding evidence that reassortment or recombination occurred on a large scale, few isolates remain available from the 1969–1970 and 1974–1976 outbreaks in Zimbabwe and South Africa. Nevertheless, multiple new lineages were encountered during 1969–1981, particularly in Zimbabwe where intensive monitoring was instituted during 1971–1979 and where lineage A and C isolates were obtained (*37*) (Figure A1; Figure 1). Moreover, it can be deduced from reported Bayesian analysis (*6*) that emergence of new lineages and genotypes surged during this period. An alternative interpretation is that reassortment and recombination occur infrequently but that mutations result from as yet inadequately explored interactions during replication in co-infections or from the generation of neutralizing antibody–escape mutants (*38*).

Thus, the partial M segment nucleotide sequence of reassortant isolate SA184/10 differs from that of vaccine virus, and that of isolate 95EG Cow-2509 from the aborted fetus of a vaccinated cow differs even more markedly from that of vaccine virus and from all other known isolates from Egypt but has some relationship with isolates H1739 and H1825 in lineage L (Figure A1), which caused human deaths. The implication is that further investigation of evolutionary relationships in lineages K, L, and M are warranted, including whole-genome sequencing of additional isolates identified in the present study. It could be relevant that viruses attenuated through intracranial passage in mice may acquire new tissue tropisms and pathogenic properties (*39**,**40*).

New vaccines, some with natural or induced attenuating deletions or mutations in all 3 segments of the genome, are being developed (*1*). However, interactions of replication-competent strains with wild virus should be investigated, especially because some vaccines were deliberately modeled on viruses considered to be particularly virulent.

## Appendix

**Table A1:** Virus isolates used for molecular epidemiologic analysis of Rift Valley fever virus*

Isolate	Year	Country	Source	Passage	Donor†	Lineage	GenBank accession no.
**MgH824**	**1979**	**Madagascar**	**Human**	**mic6, tc4**	**1**	**A**	**HM587040‡**
**MgAr811**	**1979**	**Madagascar**	***Aedes* spp. mosquito**	**NA**	**1**	**A**	**HM587041‡**
**ZH 501**	**1977**	**Egypt**	**Human**	**mic2, tc3**	**GenBank**	**A**	**M33072‡**
**94EG Tambul**	**1994**	**Egypt**	**Ovine**	**NA**	**2**	**A**	**HM587042‡**
94EG Bahr	1994	Egypt	Bovine	NA	2	A	Seq as above
**ZS6365**	**1979**	**Egypt**	**Ovine**	**mic2, tc2**	**GenBank**	**A**	**DQ 380205‡**
ZH548	1977	Egypt	Human	mic2, tc2	GenBank	A	M33075‡
ZH1776	1978	Egypt	Human	mic2, tc2	GenBank	A	DQ 380203‡
ZM657	1978	Egypt	*Culex pipiens* mosquito	mic2, tc2	GenBank	A	DQ380204
ZC3349	1978	Egypt	Bovine	mic2, tc2	GenBank	A	DQ380206
93EG Abeer	1993	Egypt	Human	mic1, tc2	2	A	HM587043‡
93EG Buffalo	1993	Egypt	Asian buffalo	mic1, tc1	2	A	Seq as above
**MP12§**	**1977**	**Egypt**	**RVF isolate ZH548**	**NA**	**GenBank**	**A**	**DQ380208‡**
**VRL2250/74**	**1974**	**Zimbabwe**	**Bovine**	**mic3, tc2**	**GenBank**	**A**	**DQ 380209‡**
**VRL1516/78**	**1978**	**Zimbabwe**	**Ovine**	**mic2**	**3**	**A**	**HM587044‡**
VRL1750/78	1978	Zimbabwe	Ovine	mic2	3	A	Seq as above
VRL1842/78	1978	Zimbabwe	Ovine	mic2	3	A	Seq as above
**S35**	**1972**	**Kenya**	**Ovine**	**NA**	**4**	**B**	**HM587045‡**
**SPU52/99/1**	**1999**	**South Africa**	**African buffalo**	**mic2**	**5**	**C**	**HM587046‡**
**SPU52/99/5**	**1999**	**South Africa**	**African buffalo**	**mic2**	**5**	**C**	**HM587047‡**
**SPU52/99/2**	**1999**	**South Africa**	**African buffalo**	**mic2**	**5**	**C**	**HM587048‡**
**SPU52/99/3**	**1999**	**South Africa**	**African buffalo**	**mic2**	**5**	**C**	**HM587049‡**
SPU52/99/4	1999	South Africa	African buffalo	mic2	5	C	Seq as above
SPU52/99/6	1999	South Africa	African buffalo	mic2	5	C	Seq as above
SPU50/99/1	1999	South Africa	Waterbuck	mic2	5	C	Seq as above
**An278**	**2000**	**Saudi Arabia**	**Ovine**	**0**	**5**	**C**	**HM587050‡**
Ar21218	2000	Saudi Arabia	*Ae. vexans arabiensis* mosquito	0	5	C	Seq as above
Ar21219	2000	Saudi Arabia	*Ae. vexans arabiensis* mosquito	0	5	C	Seq as above
Ar21220	2000	Saudi Arabia	*Cx. tritaeniorhynus* mosquito	0	5	C	Seq as above
Ar21229	2000	Saudi Arabia	*Ae. vexans arabiensis* mosquito	0	5	C	Seq as above
Ar21233	2000	Saudi Arabia	*Ae. vexans arabiensis* mosquito	0	5	C	Seq as above
Ar21236	2000	Saudi Arabia	*Ae. vexans arabiensis* mosquito	0	5	C	Seq as above
10911	2000	Saudi Arabia	Human	tc1	GenBank	C	DQ 380197
**SA01–1322**	**2001**	**Saudi Arabia**	***Ae. vexans arabiensis* mosquito**	**tc2**	**GenBank**	**C**	**AF393745‡**
**SPU12/99/21**	**1998**	**Somalia**	**Goat**	**0**	**5**	**C**	**HM587051‡**
SPU65/98/1598	1998	Tanzania	Human	0	5	C	Seq as above
SPU65/98/1380	1998	Tanzania	Human	0	5	C	Seq as above
**SPU384/97/1**	**1997**	**Kenya**	**Human**	**0**	**5**	**C**	**HM587052‡**
SPU384/97/5	1997	Kenya	Human	0	5	C	Seq as above
SPU384/97/20	1997	Kenya	Human	0	5	C	Seq as above
SPU384/97/24	1997	Kenya	Human	0	5	C	Seq as above
SPU2/98/1	1998	Kenya	Human	0	5	C	HM587053‡
SPU2/98/3	1998	Somalia	Human	0	5	C	Seq as above
SPU12/98/2	1998	Somalia	Goat	0	5	C	Seq as above
VRL2241/98	1998	Zimbabwe	Bovine	mic2	3	C	Seq as above
VRL2181/98	1998	Zimbabwe	Bovine	mic2	3	C	Seq as above
VRL2104/98	1998	Zimbabwe	Ovine	mic1	3	C	Seq as above
VRL2215/98	1998	Zimbabwe	Ovine	mic2	3	C	Seq as above
**VRL2413/98**	**1998**	**Zimbabwe**	**Bovine**	**mic1**	**3**	**C**	**HM587054‡**
**SPU2/98/9**	**1998**	**Kenya**	**Goat**	**0**	**5**	**C**	**HM587055‡**
**00523**	**1998**	**Kenya**	**Human**	**tc2**	**GenBank**	**C**	**DQ 380196‡**
**VRL1187/79**	**1979**	**Zimbabwe**	**Bovine**	**mic3**	**3**	**C**	**HM587056‡**
MgAn1002	1991	Madagascar	Bovine	NA	1	C	HM587057‡
VRL1548/78	1978	Zimbabwe	Bovine	mic2	3	C	HM587059‡
VRL2142/78	1978	Zimbabwe	Ovine	mic2	3	C	Seq as above
**VRL2354/78**	**1978**	**Zimbabwe**	**Bovine**	**mic2**	**3**	**C**	**HM587058‡**
**MgAn991**	**1991**	**Madagascar**	**Bovine**	**NA**	**1**	**C**	**HM587060‡**
**MgAn990**	**1991**	**Madagascar**	**Bovine**	**NA**	**1**	**C**	**HM587061‡**
**SPU22/07/118**	**2007**	**Kenya**	**Human**	**0**	**5**	**C**	**HM587062‡**
**SPU22/07/125**	**2007**	**Kenya**	**Human**	**0**	**5**	**C**	**HM587063‡**
**SPU22/07129**	**2007**	**Kenya**	**Human**	**0**	**5**	**C**	**HM587064‡**
SPU103/07/1	2007	Kenya	Human	0	5	C	Seq as above
SPU103/07/2	2007	Kenya	Human	0	5	C	Seq as above
SPU103/07/14	2007	Kenya	Human	0	5	C	Seq as above
SPU103/07/35	2007	Kenya	Human	0	5	C	Seq as above
H1MAU03	2003	Mauritania	Human	0	GenBank	C	EF160116‡
SPU86/09	2009	South Africa	Human	0	5	C	HM587065‡
SPU89/09	2009	South Africa	Human	0	5	C	Seq as above
SA72/09	2009	South Africa	Human	0	5	C	Seq as above
SA77/09	2009	South Africa	Human	0	5	C	Seq as above
SA152/08	2008	South Africa	Human	0	5	C	HM587066‡
M84/08	2008	South Africa	Bovine	0	5	C	Seq as above
**M48**	**2008**	**Madagascar**	**Bovine**	**NA**	**GenBank**	**C**	**HQ009512‡**
**H2MAU03**	**2003**	**Mauritania**	**Human**	**0**	**GenBank**	**C**	**EF160115‡**
**M37/08**	**2008**	**South Africa**	**African buffalo**	**0**	**6**	**C**	**HM587067‡**
**SPU22/07/4**	**2007**	**Kenya**	**Human**	**0**	**5**	**C**	**HM587068‡**
SPU22/07/126	2007	Kenya	Human	0	5	C	Seq as above
SPU22/07/120	2007	Kenya	Human	0	5	C	Seq as above
SPU22/07/121a	2007	Kenya	Human	0	5	C	Seq as above
SPU22/07/121b	2007	Kenya	Human	0	5	C	Seq as above
**SA52/08**	**2008**	**South Africa**	**Human**	**0**	**5**	**C**	**HM587069‡**
J40/6	2008	South Africa	African buffalo	0	6	C	Seq as above
J48/6	2008	South Africa	African buffalo	0	6	C	Seq as above
N2131	2008	South Africa	African buffalo	0	6	C	Seq as above
**SPU204/85**	**1985**	**Angola**	**Human**	**mic2**	**5**	**C**	**HM587076‡**
**B309**	**1977**	**Kenya**	**Bovine**	**NA**	**4**	**C**	**HM587070‡**
**VRL825/79**	**1979**	**Zimbabwe**	**Bovine**	**mic3**	**3**	**C**	**HM587071‡**
VRL1217/78	1978	Zimbabwe	Ovine	mic2	3	C	HM587073‡
VRL845/78	1978	Zimbabwe	Bovine	mic2	3	C	Seq as above
VRL2129/78	1978	Zimbabwe	Bovine	mic2	3	C	Seq as above
VRL688/78	1978	Zimbabwe	Bovine	mic1	3	C	Seq as above
**VRL2051/76**	**1976**	**Zimbabwe**	**Human**	**mic3**	**3**	**C**	**HM587072‡**
**21445**	**1983**	**Kenya**	***Ae. mcintoshi* mosquito**	**ha1, tc1**	**7**	**C**	**HM587074‡**
B1143	1977	Kenya	Bovine	NA	4	C	HM587075‡
**VRL1290/78**	**1978**	**Zimbabwe**	**Bovine**	**mic2**	**3**	**C**	**HM587077‡**
VRL1508/78	1978	Zimbabwe	Bovine	mic2	3	C	Seq as above
**73HB1230**	**1973**	**CAR**	**Human**	**mic6, tc4**	**GenBank**	**D**	**DQ 380221‡**
**VRL2140A/75**	**1975**	**Zimbabwe**	**Bovine**	**mic2**	**3**	**E**	**HM587078‡**
**SPU44/85**	**1985**	**Zambia**	**Human**	**mic1, tc3**	**5**	**E**	**HM587079‡**
SPU45/85	1985	Zambia	Human	mic1, tc3	5	E	Seq as above
**VRL1887/78**	**1978**	**Zimbabwe**	**Bovine**	**mic2**	**3**	**E**	**HM587080‡**
VRL1905/78	1978	Zimbabwe	Bovine	mic2	3	E	Seq as above
VRL3108/78	1978	Zimbabwe	Bovine	mic2	3	E	Seq as above
VRL2230/78	1978	Zimbabwe	Bovine	mic2	3	E	Seq as above
**VRL1260/78**	**1978**	**Zimbabwe**	**Bovine**	**mic2**	**3**	**E**	**HM587081‡**
**74HB59**	**1974**	**CAR**	**Human**	**mic6, tc3**	**7**	**E**	**HM587082‡**
73HB1449	1973	CAR	Human	mic5, tc1	GenBank	E	DQ 380211‡
**Ar20364**	**1981**	**South Africa**	***Cx. zombaensis* mosquito**	**mic4**	**5**	**F**	**HM587101‡**
Ar20368	1981	South Africa	*Cx. zombaensis* mosquito	mic3	5	F	Seq as above
**ArB1976 (Zinga)**	**1969**	**CAR**	***Mansonia africana* mosquito**	**mic5, tc3**	**7**	**G**	**HM587083‡**
**AnK6087**	**1984**	**Guinea**	***Micropteropus pusillus* bat**	**NA**	**GenBank**	**G**	**AF134502‡**
**AnK3837**	**1981**	**Guinea**	***Hipposideros caffer* bat**	**mic7, tc1**	**7**	**G**	**HM587084‡**
**ArD104769**	**1993**	**Senegal**	***Ae. vexans arabiensis* mosquito**	**NA**	**GenBank**	**G**	**AF134499‡**
**VRL1032/78**	**1978**	**Zimbabwe**	**Bovine**	**mic2**	**3**	**G**	**HM587085‡**
VRL1853/78	1978	Zimbabwe	Bovine	mic2	3	G	
**HvB375**	**1985**	**CAR**	**Human**	**mic4, tc4**	**GenBank**	**G**	**DQ 380218‡**
**CAR R1662**	**1985**	**CAR**	**Human**	**mic4, tc3**	**7**	**G**	**HM587086‡**
**CAR R1752**	**1986**	**CAR**	**Human**	**mic4, tc3**	**7**	**G**	**HM587087‡**
**SA71/10**	**2010**	**South Africa**	**Human**	**0**	**5**	**H**	**HM587088‡**
**SA482/10**	**2010**	**South Africa**	**Human**	**0**	**5**	**H**	**HM587089‡**
**SA1221/10**	**2010**	**South Africa**	**Human**	**0**	**5**	**H**	**HM587090‡**
**SA59/10**	**2010**	**South Africa**	**Human**	**0**	**5**	**H**	**HM587091‡**
**SA54/10**	**2010**	**South Africa**	**Human**	**0**	**5**	**H**	**HM587092‡**
SA79/10	2010	South Africa	Human	0	5	H	Seq as above
SA122/10	2010	South Africa	Human	0	5	H	Seq as above
SA197/10	2010	South Africa	Human	0	5	H	Seq as above
SA226/10	2010	South Africa	Human	0	5	H	Seq as above
SA227/10	2010	South Africa	Human	0	5	H	Seq as above
SA257/10	2010	South Africa	Human	0	5	H	Seq as above
SA360/10	2010	South Africa	Human	0	5	H	Seq as above
SA377/10	2010	South Africa	Human	0	5	H	Seq as above
SA420/10	2010	South Africa	Human	0	5	H	Seq as above
SA486/10	2010	South Africa	Human	0	5	H	Seq as above
SA509/10	2010	South Africa	Human	0	5	H	Seq as above
SA533/10	2010	South Africa	Human	0	5	H	Seq as above
SA578/10	2010	South Africa	Human	0	5	H	Seq as above
SA591/10	2010	South Africa	Human	0	5	H	Seq as above
SA611/10	2010	South Africa	Human	0	5	H	Seq as above
SA663/10	2010	South Africa	Human	0	5	H	Seq as above
SA1143/10	2010	South Africa	Human	0	5	H	Seq as above
SA1147/10	2010	South Africa	Human	0	5	H	Seq as above
SA1171/10	2010	South Africa	Human	0	5	H	Seq as above
SA1325/10	2010	South Africa	Human	0	5	H	Seq as above
**SA276/10**	**2010**	**South Africa**	**Human**	**0**	**5**	**H**	**HM587093‡**
**SA106/10**	**2010**	**South Africa**	**Human**	**0**	**5**	**H**	**HM587094‡**
SA100/10	2010	South Africa	Human	0	5	H	Seq as above
SA195/10	2010	South Africa	Human	0	5	H	Seq as above
SA225/10	2010	South Africa	Human	0	5	H	Seq as above
SA325/10	2010	South Africa	Human	0	5	H	Seq as above
SA328/10	2010	South Africa	Human	0	5	H	Seq as above
SA492/10	2010	South Africa	Human	0	5	H	Seq as above
**SA423/10**	**2010**	**South Africa**	**Human**	**0**	**5**	**H**	**HM587095‡**
**SA404/10**	**2009**	**South Africa**	**Human**	**0**	**5**	**H**	**HM587096‡**
**SA373/10**	**2010**	**South Africa**	**Human**	**0**	**5**	**H**	**HM587097‡**
**SA85/10**	**2010**	**South Africa**	**Human**	**0**	**5**	**H**	**HM587098‡**
SPU68/10	2010	South Africa	Human	0	5	H	Seq as above
SA95/10	2010	South Africa	Human	0	5	H	Seq as above
SA206/10	2010	South Africa	Human	0	5	H	Seq as above
SA317/10	2010	South Africa	Human	0	5	H	Seq as above
SA394/10	2010	South Africa	Human	0	5	H	Seq as above
SA395/10	2010	South Africa	Human	0	5	H	Seq as above
SA414/10	2010	South Africa	Human	0	5	H	Seq as above
SA1108/10	2010	South Africa	Human	0	5	H	Seq as above
**SA1224/10**	**2010**	**South Africa**	**Human**	**0**	**5**	**H**	**HM587099‡**
**SPU77/04**	**2004**	**Namibia**	**Human**	**0**	**5**	**H**	**HM587100‡**
**An1830**	**1956**	**South Africa**	**Ovine**	**mic5**	**5**	**I**	**HM587108‡**
**Ar74**	**1955**	**South Africa**	***Ae. circumluteolus* mosquito**	**mic7**	**5**	**I**	**HM587109‡**
**VRL2269/74**	**1974**	**Zimbabwe**	**Bovine**	**mic2**	**GenBank**	**J**	**DQ 380222‡**
**SNS¶**	**1944**	**Uganda**	***Eretmapodites* spp. bat**	**mic103, tc2**	**8**	**K**	**HM587102‡**
SNS 105/2010#	1944	Uganda	*Eretmapodites* spp. bat	mic103, tc5	8	K	Seq as above
95EG vaccine**	1944	Uganda	RVF strain SNS	NA	2	K	HM587103‡
KEN57 Rintoul	1951	Kenya	Ovine	mic31, tc1	7	K	HM587104‡
**Entebbe/KCS††**	**1944**	**Uganda**	***Eretmapodites* spp. bat**	**mip184, tc2**	**GenBank**	**K**	**DQ380191‡**
**B314**	**1963**	**Kenya**	**Bovine**	**NA**	**4**	**K**	**HM587105‡**
**B674**	**1962**	**Kenya**	**Bovine**	**NA**	**4**	**K**	**HM587106‡**
**SA184/10**	**2010**	**South Africa**	**Human**	**0**	**5**	**K**	**HM587107‡**
**VRL2373/74**	**1974**	**Zimbabwe**	**Bovine**	**mic3**	**3**	**K**	**HM587121‡**
**H1739**	**1975**	**South Africa**	**Human**	**NA**	**5**	**L**	**HM587110‡**
**VRL763/70**	**1970**	**Zimbabwe**	**Bovine**	**mic2**	**3**	**L**	**HM587111‡**
**Ar12568**	**1971**	**South Africa**	***Er. quinquivittatus* bat**	**mic3**	**5**	**L**	**HM587112‡**
**Ar11186**	**1969**	**Zimbabwe**	***Ae. lineatopennis* mosquito**	**mic5**	**5**	**L**	**HM587113‡**
**H1825**	**1975**	**South Africa**	**Human**	**NA**	**5**	**L**	**HM587114‡**
35/74	1975	South Africa	Bovine	NA	GenBank	L	JF784387‡
**95EG Cow-2509**	**1995**	**Egypt**	**Bovine**	**NA**	**2**	**L**	**HM587115‡**
**KEN56/B2653/IB8**	**1963**	**Kenya**	**Bovine**	**ha1, mic8, tc27**	**4**	**L**	**HM587117‡**
Kitale 1840	1964	Kenya	Bovine	NA	4	L	HM587116‡
**SA75**	**1975**	**South Africa**	**Human**	**tc3**	**GenBank**	**L**	**DQ380189‡**
**Lunyo**	**1955**	**Uganda**	***Aedes* spp. mosquito**	**mic3**	**5**	**M**	**HM587119‡**
**Ar118**	**1955**	**South Africa**	***Ae. circumluteolus* mosquito**	**mic7**	**5**	**M**	**HM587120‡**
**OS-4**	**1988**	**Mauritania**	**Human**	**tc2**	**7**	**N**	**HM587122‡**
OS-1	1987	Mauritania	Human	tc2	GenBank	N	DQ380186
OS-3	1987	Mauritania	Human	tc2	GenBank	N	DQ380184
OS-8	1987	Mauritania	Human	tc2	GenBank	N	DQ380185
OS-9	1987	Mauritania	Human	tc2	GenBank	N	DQ380183
**HD47502**	**1987**	**Mauritania**	**Human**	**NA**	**GenBank**	**N**	**AF134494‡**
**HD47311**	**1987**	**Mauritania**	**Human**	**NA**	**GenBank**	**N**	**AF134495‡**
**ArD38388**	**1983**	**Burkina Faso**	***Ae. cuminsi* mosquito**	**tc5**	**GenBank**	**N**	**DQ380187‡**
**ArD38457**	**1984**	**Burkina Faso**	***Ae. furcifer* mosquito**	**NA**	**GenBank**	**N**	**AF134497‡**
**HD47408**	**1987**	**Mauritania**	**Human**	**NA**	**GenBank**	**N**	**AF134496‡**
**HD21955**	**1975**	**Senegal**	**Human**	**mic5**	**7**	**N**	**HM587123‡**
**ArD38661**	**1983**	**Senegal**	***Ae. dalzieli* mosquito**	**NA**	**7**	**N**	**HM587124‡**
**AnD106417**	**1993**	**Senegal**	**Bovine**	**NA**	**GenBank**	**N**	**AF134500‡**
**SA51 van Wyk**	**1951**	**South Africa**	**Ovine**	**ov3, mic2**	**5**	**O**	**HM587125‡**

*mic, mouse intracranial; tc, tissue culture; NA, not available; seq, sequence; RVF, Rift Valley fever; ha, hamster; CAR, Central African Republic; SNS, Smithburn neurotropic strain; mip, mouse intraperitoneal; ov, ovine. Isolates are listed in order of appearance in phylogenetic groups in the tree in Figure A1. Ninety-five isolates displaying unique sequences are indicated in **boldface**. Isolates in standard text have identical sequences to isolates indicated in **boldface** directly above. †1, Institut Pasteur, Antananarivo, Madagascar; 2, US Naval Medical Research Unit 3, Cairo, Egypt; 3, Central Veterinary Research Laboratory, Harare, Zimbabwe; 4, Central Veterinary Research Laboratory, Nairobi, Kenya; 5, National Institute for Communicable Diseases, Johannesburg, South Africa; 6, Agricultural Research Council–Onderstepoort Veterinary Institute, Pretoria, South Africa; 7, US Army Medical Research Institute for Infectious Diseases, Frederick, MD, USA; 8, Onderstepoort Biologic Products, Pretoria, South Africa. ‡Sequences selected for analysis and inclusion in the tree (see Materials and Methods). §Candidate vaccine strain derived by culturing isolate ZH548 from Egypt with the mutagen 5-fluorouracil. ¶SNS master seed stock derived from a 1944 mosquito isolate from Uganda by serial intracranial passage of infected brain suspension in mice and used to produce live animal vaccine in BHK cell cultures. #Batch of live SNS animal vaccine from South Africa issued in 2010. **Animal vaccine from Egypt derived from SNS vaccine from South Africa. ††Hepatotropic strain derived from the same 1944 mosquito isolate from Uganda used to produce the SNS strain, but produced by serial intraperitoneal passage of infected liver suspension in mice; referred to as the Entebbe strain in some publications.
